# Supplementary material for: Polo-like kinase 1 coordinates biosynthesis during cell cycle progression by directly activating pentose phosphate pathway
Source: Nat Commun. 2017 Nov 15;8:1506. doi: 10.1038/s41467-017-01647-5 (PMC5686148; doi:10.1038/s41467-017-01647-5)
Supplement: Supplementary file 1 — Supplementary Information [file 41467_2017_1647_MOESM1_ESM.pdf]

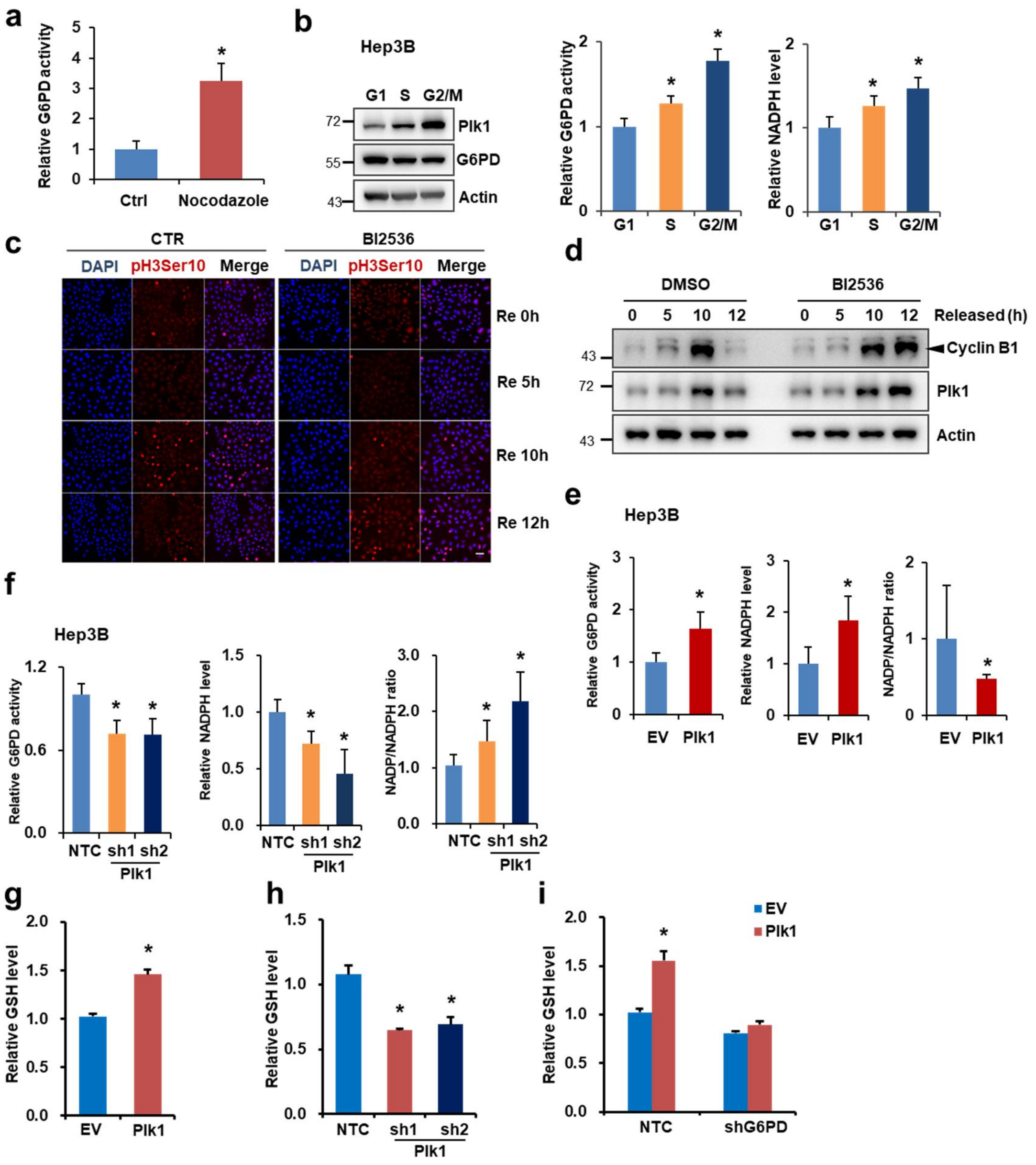

Supplementary Figure 1. Plk1 activates G6PD during cell cycle progression. (**a**) G6PD enzyme activity in HeLa cell in the presence or absence of 1000 nM nocodazole. Data were presented as mean (±SD). *p<0.05 as compared to control group by two-sided student's t-test. (**b**) Hep3B cells were synchronized at G1, S and G2/M phases using HU double block, followed by western blot and G6PD enzyme activity measurement, NADPH measurement. n=3 biologically independent replicates. Data were presented as mean (±SD). *p<0.05 as compared to G1 group by two-sided student's t-test. (**c-d**) HeLa cells were synchronized into G1 phase and released to fresh DMEM medium for 0, 5, 10, 12 hours. BI2536 or DMSO was added 1 hour before

releasing. Cell were harvested and subjected to immunofluorescence stain using anti-pH3Ser10 antibody (scale bar=50μm) (**c**) and western blot using anti-Cyclin B1 antibody (**d**). (**e**) G6PD enzyme activity, NADPH and $NADP^+$/NADPH ratio level were measured in Hep3B cells stably overexpressing Plk1. Data were presented as mean (±SD). *$p<0.05$ as compared to EV group by two-sided student's t-test. (**f**) G6PD enzyme activity, NADPH and $NADP^+$/NADPH ratio levels were measured in Hep3B cells stably expressing shPlk1. Data were presented as mean (±SD). *$p<0.05$ as compared to NTC group by two-sided student's t-test. (**g-i**) GSH levels were measured in HeLa cells overexpressing Plk1 (**g**), or expressing shPlk1 (**h**) or in Plk1-overexpressing HeLa cells with further G6PD knockdown (**i**). n=3 biologically independent replicates. Data were presented as mean (±SD). *$P<0.05$ as compared to EV or NTC group by two-sided student's t-test.

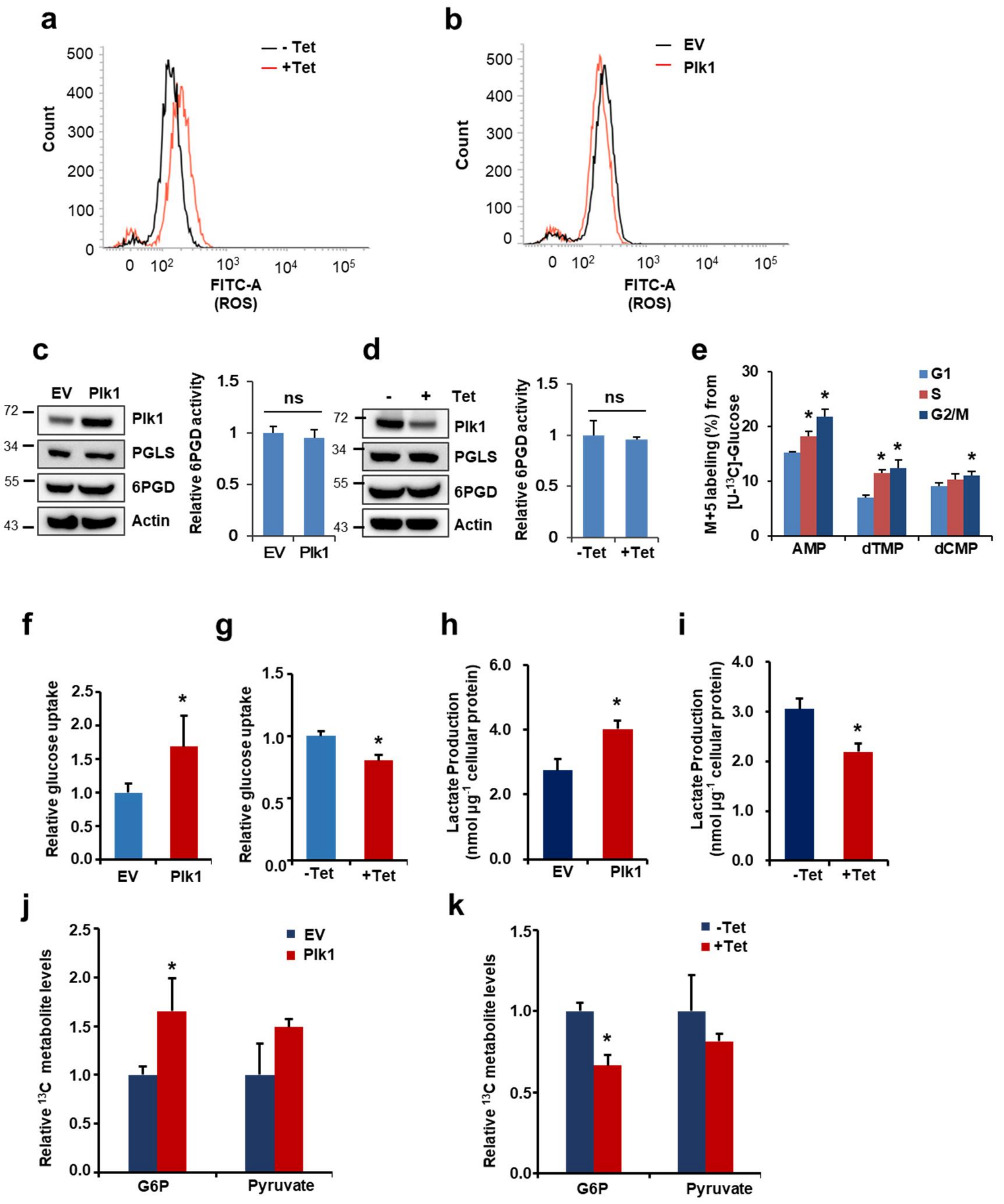

Supplementary Figure 2. Plk1 enhances pentose phosphate pathway (PPP) and biosynthesis in cancer cells. (**a-b**) ROS levels were measured in HeLa cells expressing tet-inducible shPlk1 (**a**) or in HeLa cells overexpressing Plk1 (**b**). (**c-d**) HeLa cells with Plk1 overexpression (**c**) or with Plk1 knockdown by tet-inducible shRNAs (**d**) were harvested, followed by western blot and 6PGD activity measurement. n=3 biologically independent replicates. Data were presented as mean (±SD). *p<0.05 as compared to EV or –tet group by two-sided student's t-test. ns: not significant. (**e**) HeLa cells were synchronized by double HU block which was described in Methods section. When the

cells were treated for the second round of HU block for 11 hours, i.e. 1 hour before releasing, U-^{13}C glucose was added into the cell culture medium and further culture for 2 hour and then harvested for G1 phase cell analysis. For cell samples of S and G2/M phases, after the regular second round of HU block for 12 hours, wells were released into fresh HU-free medium and further cultured for 4 hours and 9 hours, respectively, followed by supplementation of U-^{13}C glucose and 2 hour culture before harvesting cells for analysis. Cells were harvested and ^{13}C-incorporated nucleotides generated from the PPP flux were detected by LC-MS. Data were presented as mean (±SD). *P<0.05 as compared to corresponding G1 group by two-sided student's t-test. (**f-i**) Glucose uptake or overall lactate production was measured in HeLa cells overexpressing Plk1 (**f, h**) or expressing tet-inducible shPlk1 (**g, i**). Data were presented as mean (±SD). *P<0.05 as compared to corresponding EV or –tet group by two-sided student's t-test. (**j-k**) ^{13}C- incorporated G6P and pyruvate were measured in 2-^{13}C glucose metabolic flux assay by NMR in HeLa cells overexpressing Plk1 (**j**) or expressing tet-inducible shPlk1 (**k**). Data were presented as mean (±SEM). *P<0.05 as compared to corresponding EV or –tet group by two-sided student's t-test.

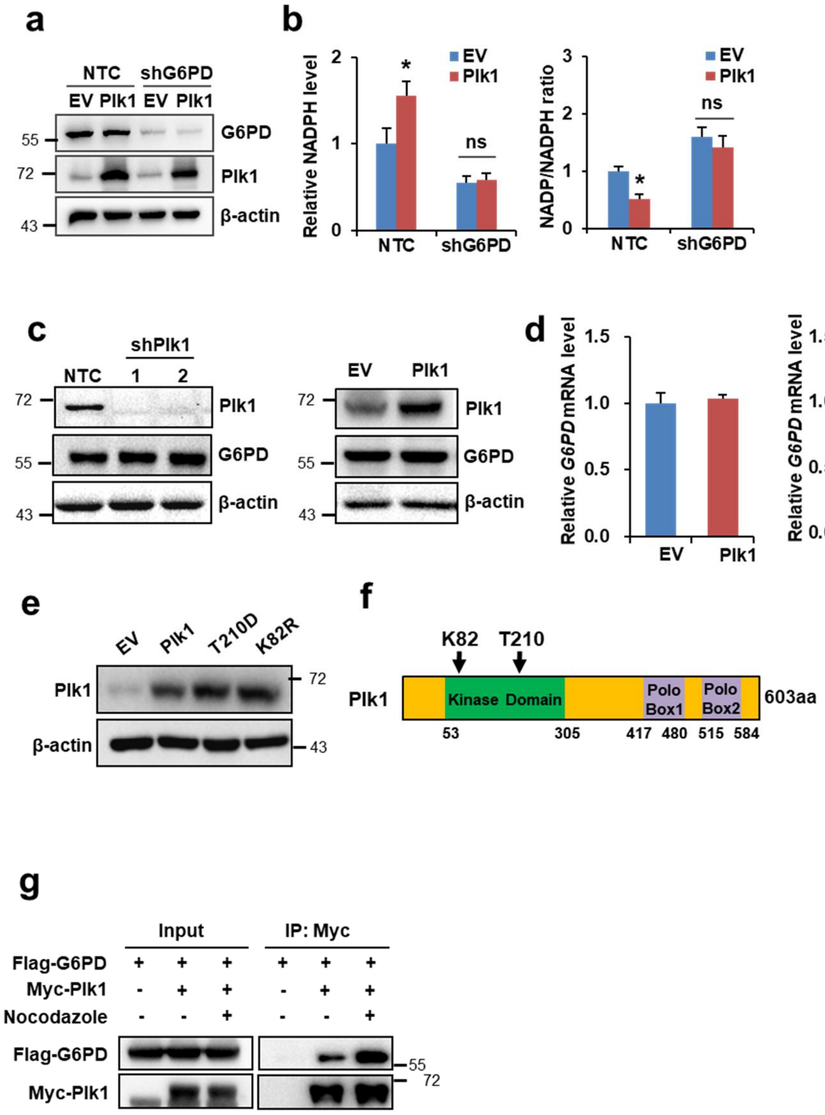

Supplementary Figure 3. Plk1 regulates G6PD activity by interacting with G6PD. (**a-b**) NTC or shG6PD expressing Hep3B cells were infected with virus expressing empty vector (EV) or vector expressing Plk1. Cells were harvested and subjected to western blot (**a**), NADPH and $NADP^+$/NADPH ratio measurements (**b**). n=3 biologically independent replicates. Data were presented as mean (±SD). *P<0.05 as compared to corresponding NTC or EV group by two-sided student's t-test. β-actin served as loading control. (**c-d**) Western blot or qRT-PCR analysis of G6PD protein (**c**) or *G6PD* mRNA (**d**) in HeLa cells expressing shPlk1 or overexpressing Plk1. n=3 biologically independent replicates, Data were presented as mean (±SD). *P<0.05 as compared to corresponding NTC or EV group by two-sided student's t-test. β-actin served as loading control. (**e**) Western blot confirming the stable expression of wild-

type Plk1 and its mutants in HeLa cells. β-actin served as loading control. (**f**) Kinase domain and Polo Box domains of human Plk1 protein. (**g**) 293T cells co-transfected with Flag-G6PD and Myc-Plk1 were treated with or without 1000 nM nocodazole. Cell lysates were immunoprecipitated with anti-myc antibody, followed by western blot analysis with antibodies against Flag or Plk1.

a

Peptide Sequence	Modifications	Protein Modifications
TKKPGMFFNPEESELDLTYGNR	Phospho(T)@1	Phospho(T)@406
CISEVQANNVVLGQYVGNPDGEGEATK	Phospho(S)@3	Phospho(S)@296

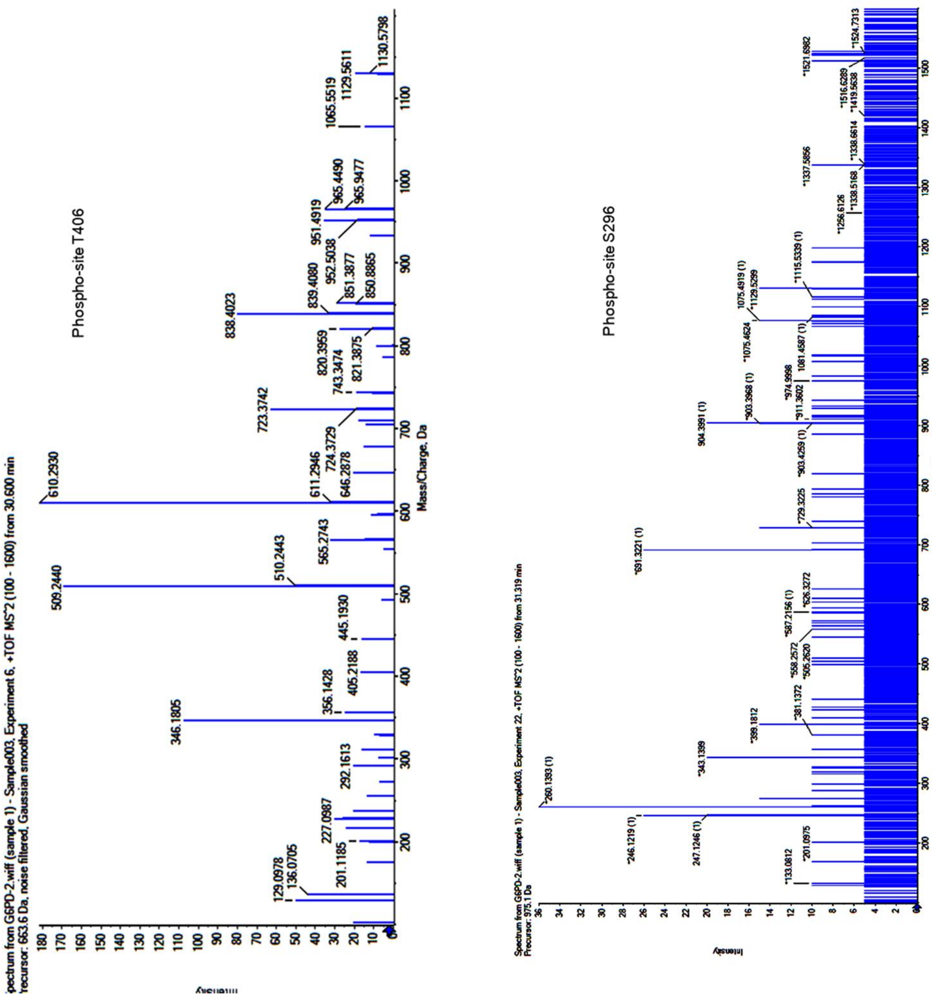

b

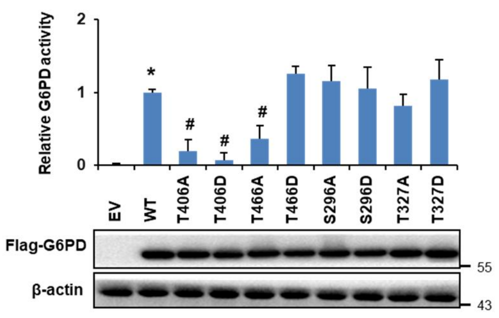

Supplementary Figure 4. Plk1 regulates G6PD activity by phosphorylating G6PD. (**a**) The phosphorylation sites (T406, S296) of Plk1 on G6PD were identified by mass spectrometry. (**b**) The G6PD enzyme activity and protein levels were measured in 293T

cells transfected with EV, wild-type G6PD or its mutants. Data were presented as mean (±SD). * $P<0.05$ as compared to EV group. # $P<0.05$ as compared to WT group. β-actin served as loading control.

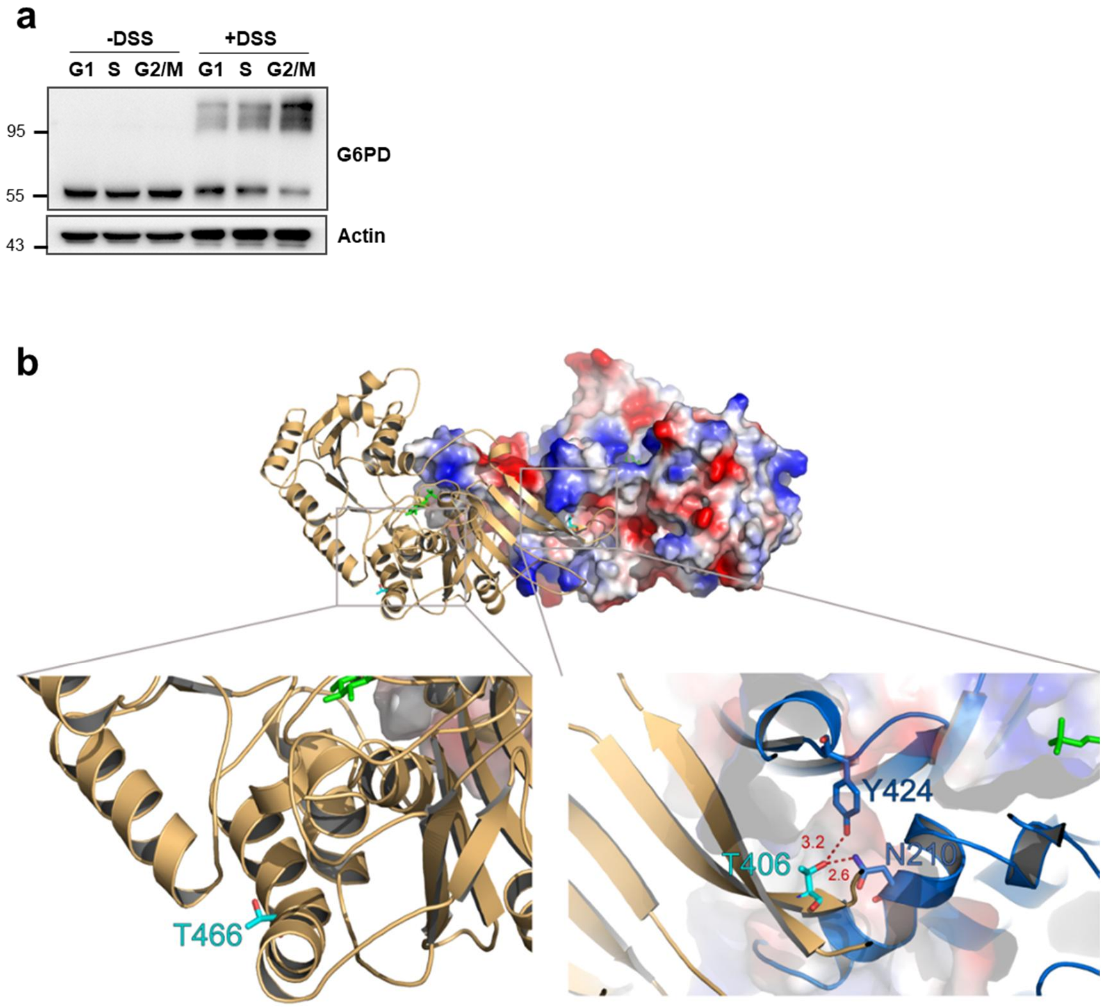

Supplementary Figure 5. The stereostructure of dimeric G6PD and the locations of its T406 and T466. (**a**) Hep3B cells were synchronized at G1, S and G2/M phases. Cells were harvested and cross-linked using 1 mM DSS, followed by western blotting with anti-G6PD. β-actin served as loading control. (**b**) The structure of G6PD dimer with the substrate G6P (PDB ID: 2BHL) generated by the PyMOL Molecular Graphics System, Version 1.7.4. (www.pymol.org). In the upper panel, one G6PD molecule is shown in cartoon representation and the other is depicted in electrostatic potential surface. G6P is labeled in green color. T406 and T466 are shown in blue color. Lower left, a detailed view of T466 residue. Shown as the lower right panel, T406 interacts with Y424 and N210 (blue sticks) of the other G6PD molecule.

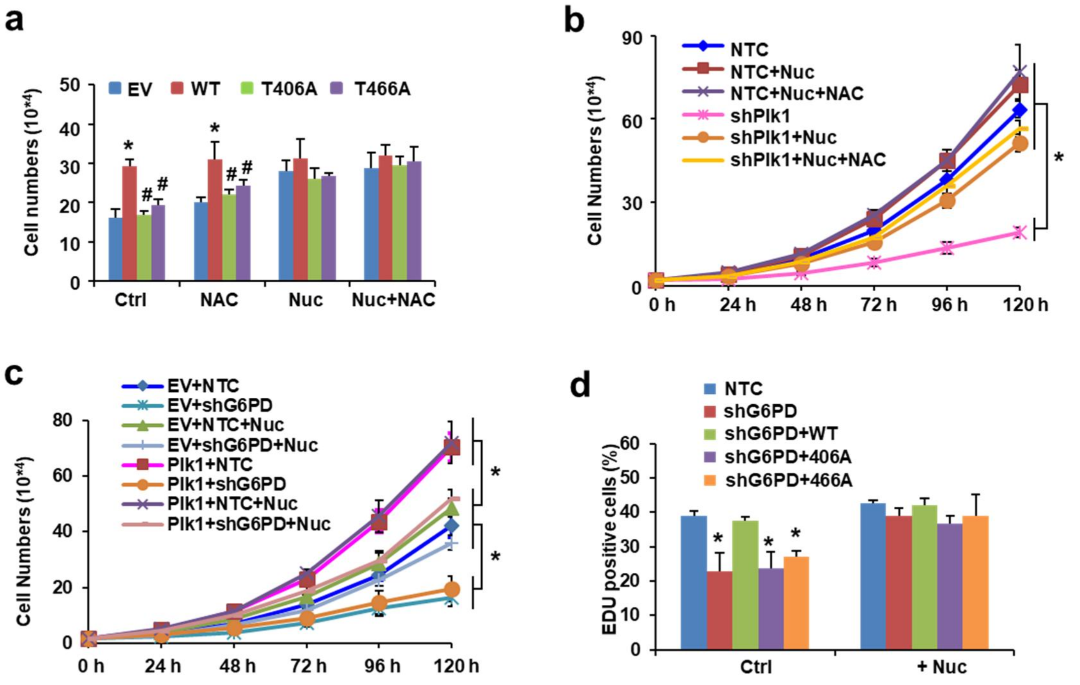

Supplementary Figure 6. G6PD-mediated PPP is vital for the cell cycle regulated by Plk1. (**a**) G6PD wild-type or its mutants were forced overexpressed in HeLa cells stably expressing shG6PD. Cells were cultured in the medium supplemented with 2 mM NAC or 25 μM nucleosides mix or both for 96 hours. Cell numbers were determined by trypan blue counting. Data were presented as mean (±SD). * P<0.05 as compared to corresponding EV group. # P<0.05 as compared to corresponding WT group. (**b**) HeLa cells stably expressing NTC or tet-inducible shPlk1 were cultured in the medium supplemented with 25 μM nucleosides mix alone or together with 2 mM NAC for indicated hours. To induce shRNA expression, cells were grown in the presence of 0.1 μg/ml doxycycline. Cell numbers were determined by trypan blue counting. (**c**) HeLa cells stably overexpressing Plk1 were further knocked down of G6PD by shRNAs. Cells were cultured in the medium supplemented with 25 μM nucleosides mix for indicated hours. Cell numbers were determined by trypan blue counting. (**d**) G6PD wild-type or its mutants were forced overexpressed in HeLa cells stably expressing shG6PD. Cellular EdU incorporation was measured in the presence or absence of the supplementation of 25 μM nucleosides mix in medium. Data were presented as mean (±SD). * P<0.05 as compared between indicated groups or compared to corresponding NTC group.

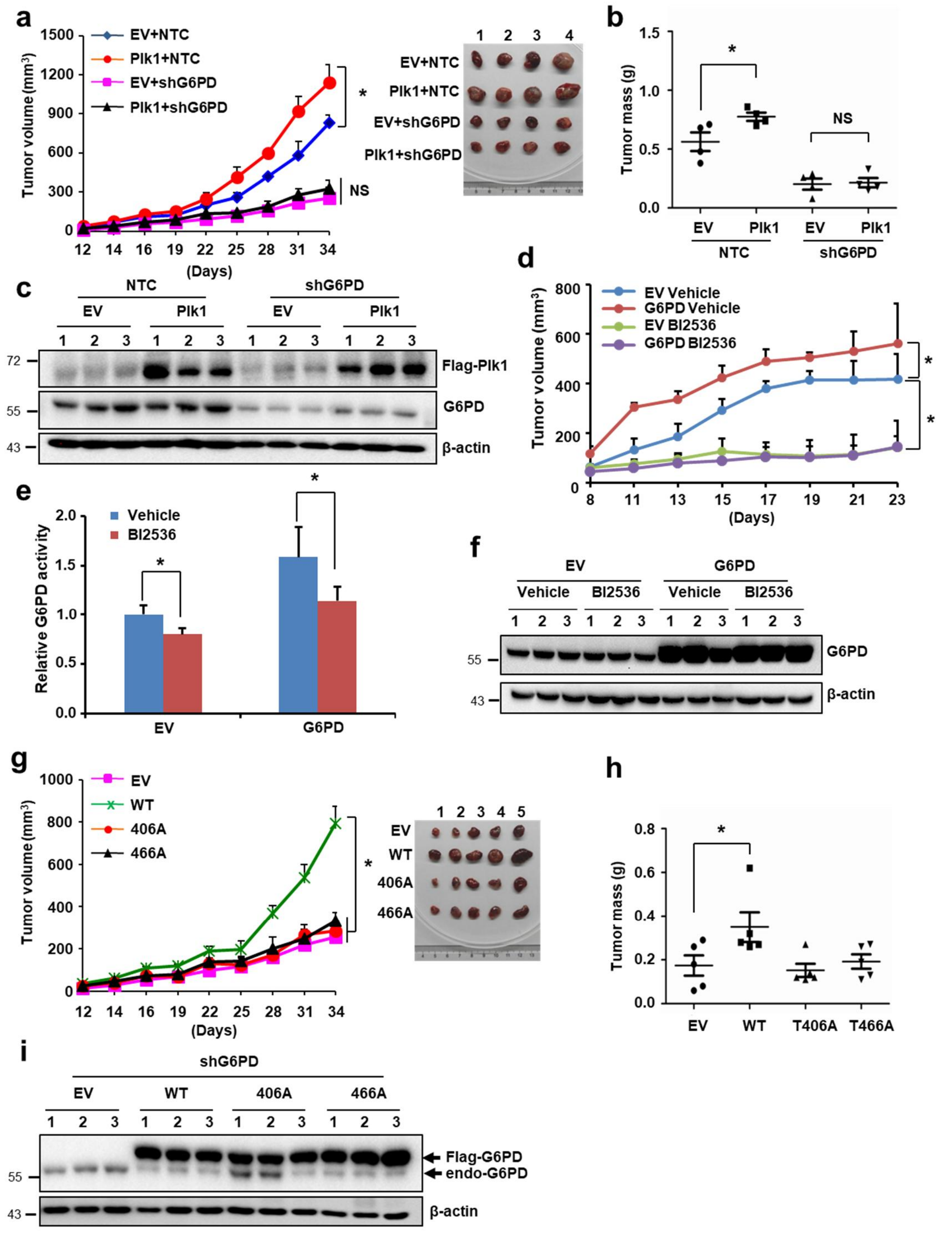

Supplementary Figure 7. G6PD is critical for Plk1-mediated tumor proliferation. (**a-c**) Hep3B cells stably expressing EV or Plk1 were further infected with viruses expressing non-targeting control (NTC) or shG6PD. Equal numbers of cells were injected subcutaneously into nude mice (n=4 for each group). Tumor growth was monitored starting from 12 days after inoculation (**a**). Tumors were extracted and compared at the end of experiment (**b**). Protein levels of Flag-Plk1 and G6PD were

determined by western blot using the lysates from tumor tissues (**c**). (**d-f**) Equal numbers of HeLa cells stably expressing empty vector (EV) or G6PD were subcutaneously injected into Balb/c nude mice (n=5 for each group). Vehicle (saline) or BI2536 (35 mg/kg body weight) were injected intraperitoneally one day before cell inoculation and subsequently twice a week. Tumor volumes were measured starting from 8 days after cell inoculation (**d**). Tumors were extracted followed by G6PD activity measurement (**e**) and protein analysis (**f**) at the end of the experiment. Data were presented as mean (±SD). *P<0.05 as compared between indicated groups by two-sided student's t-test. (**g-i**) G6PD wild-type or its mutants were forced overexpressed in Hep3B cells stably expressing shG6PD. Equal numbers of cells were injected subcutaneously into nude mice (n= 5 for each group). Tumor growth was monitored starting from 12 days after inoculation (**g**). Tumors were extracted and compared at the end of experiment (**h**). Protein levels G6PD were determined by western blot using the lysates from tumor tissues (**i**). Data were presented as mean (±SD). * P<0.05 as compared between indicated groups. β-actin served as loading control.

Fig. 1

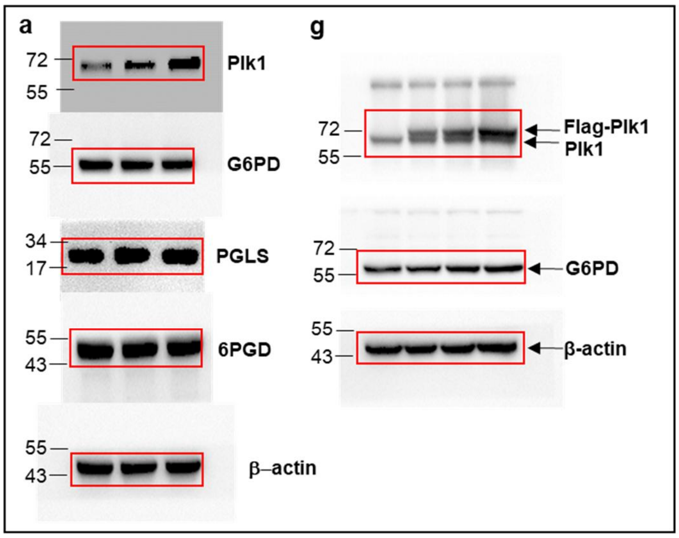

Fig. 2

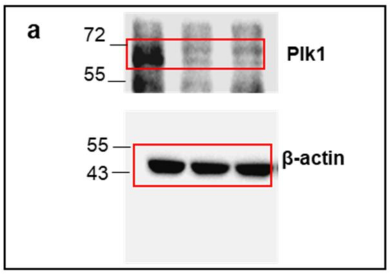

Fig. 3

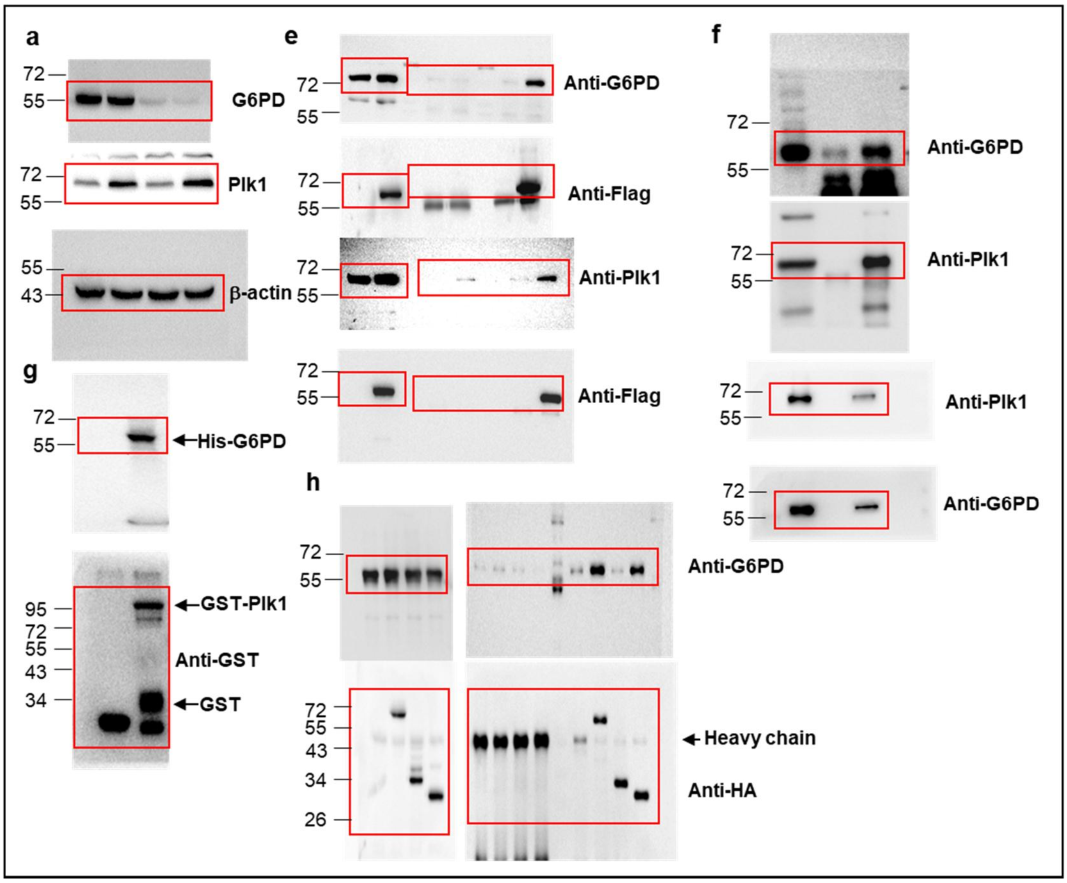

Supplementary Figure 8. The uncropped scans of immunoblots

Fig. 3

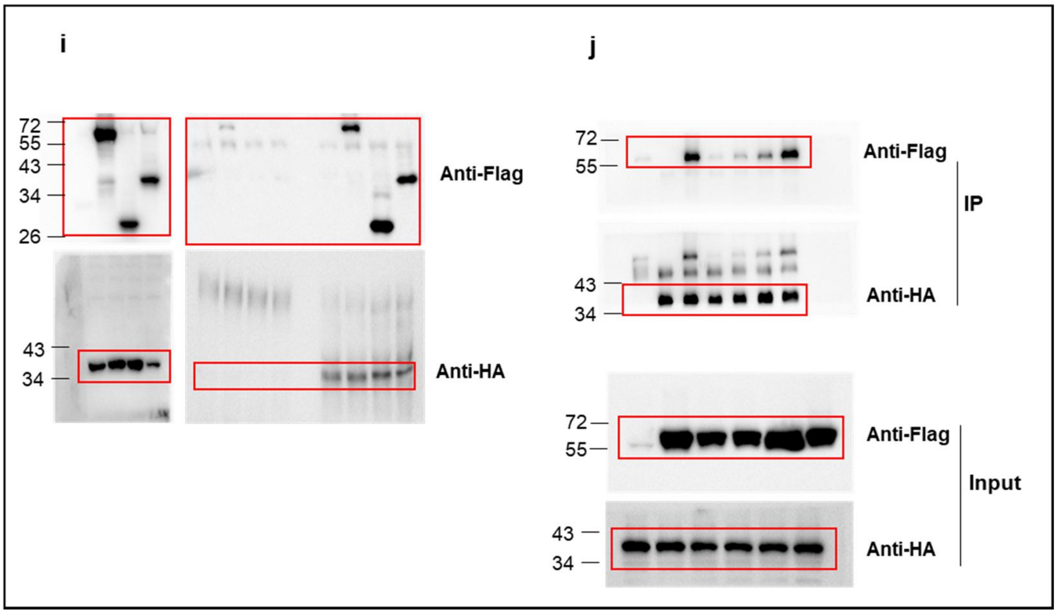

Fig. 4

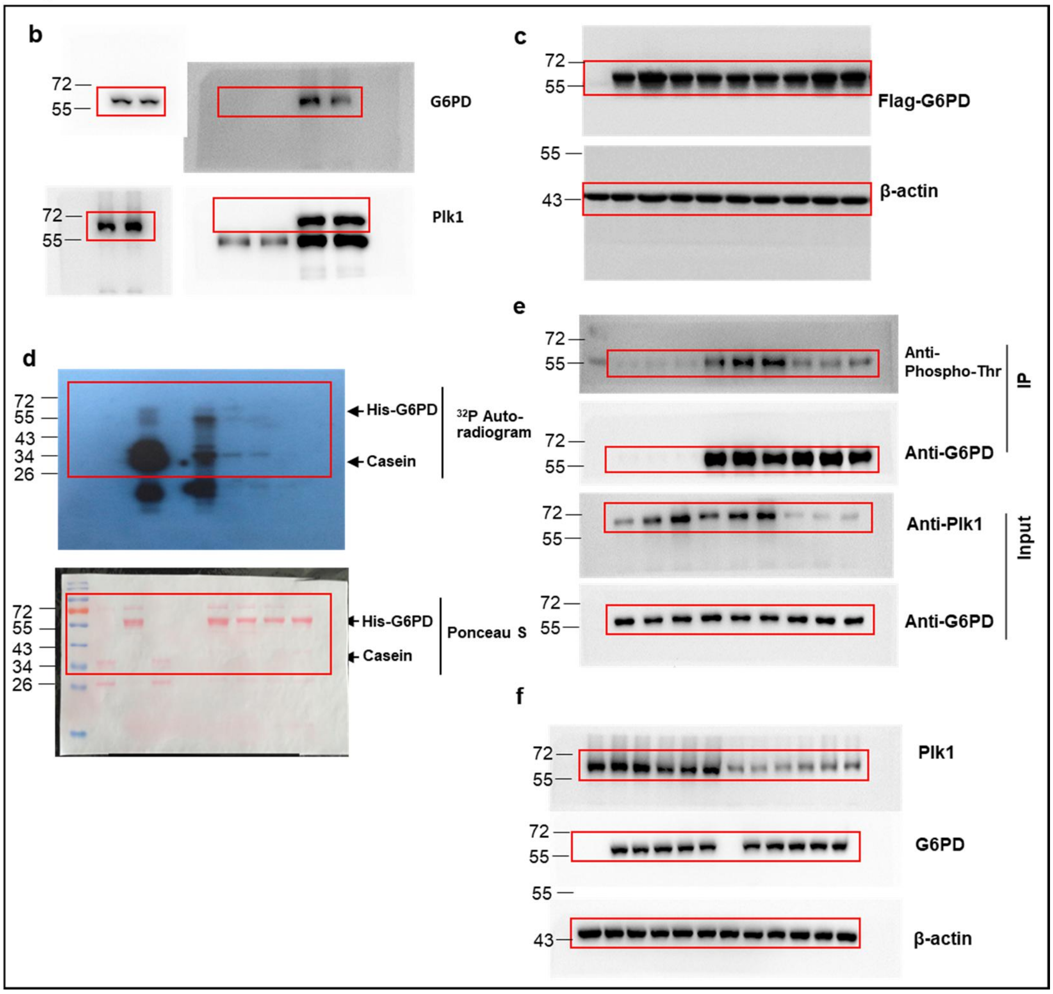

Supplementary Figure 8. The uncropped scans of immunoblots (continued).

Fig. 5

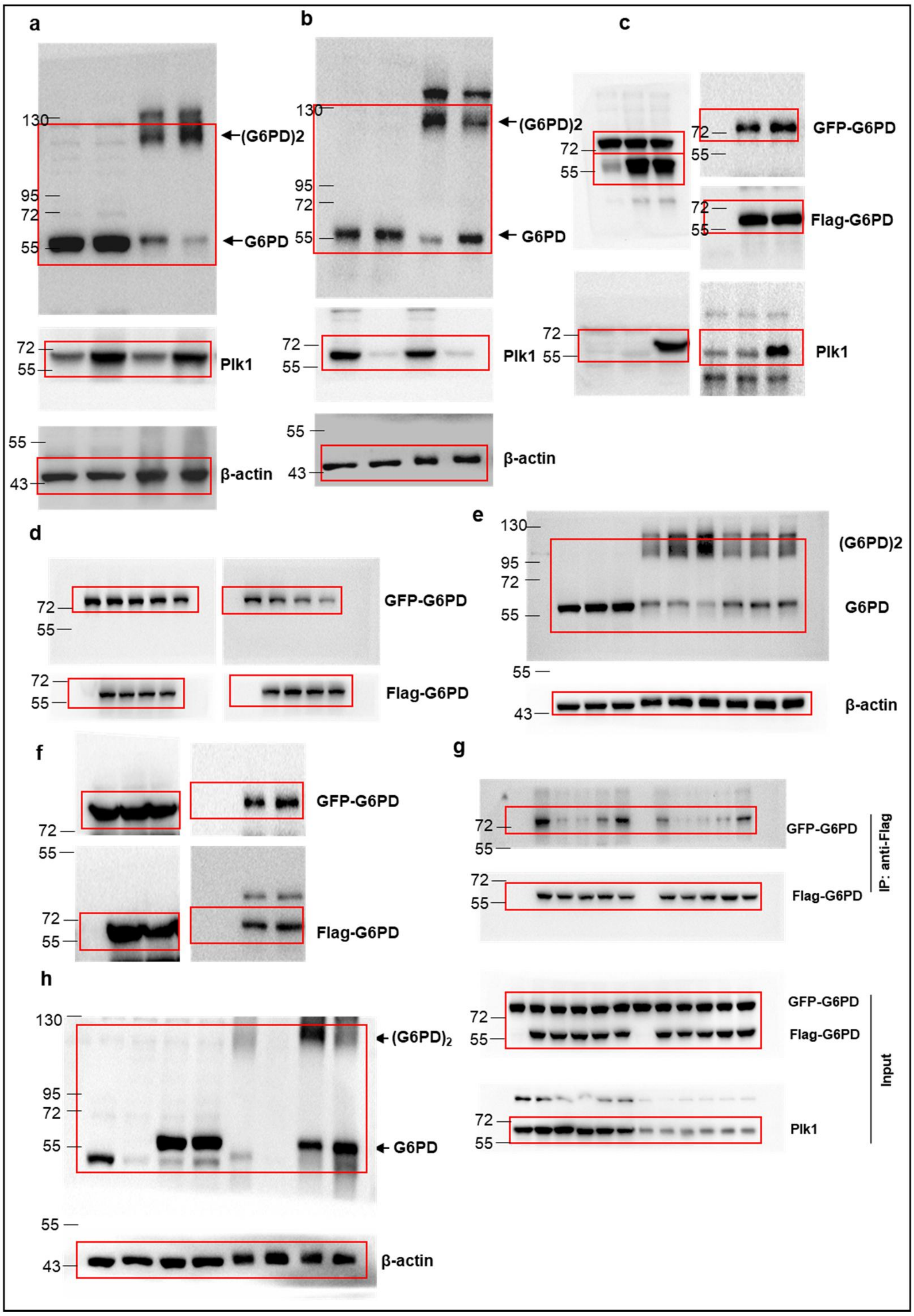

Supplementary Figure 8. The uncropped scans of immunoblots (continued).

Fig. 7

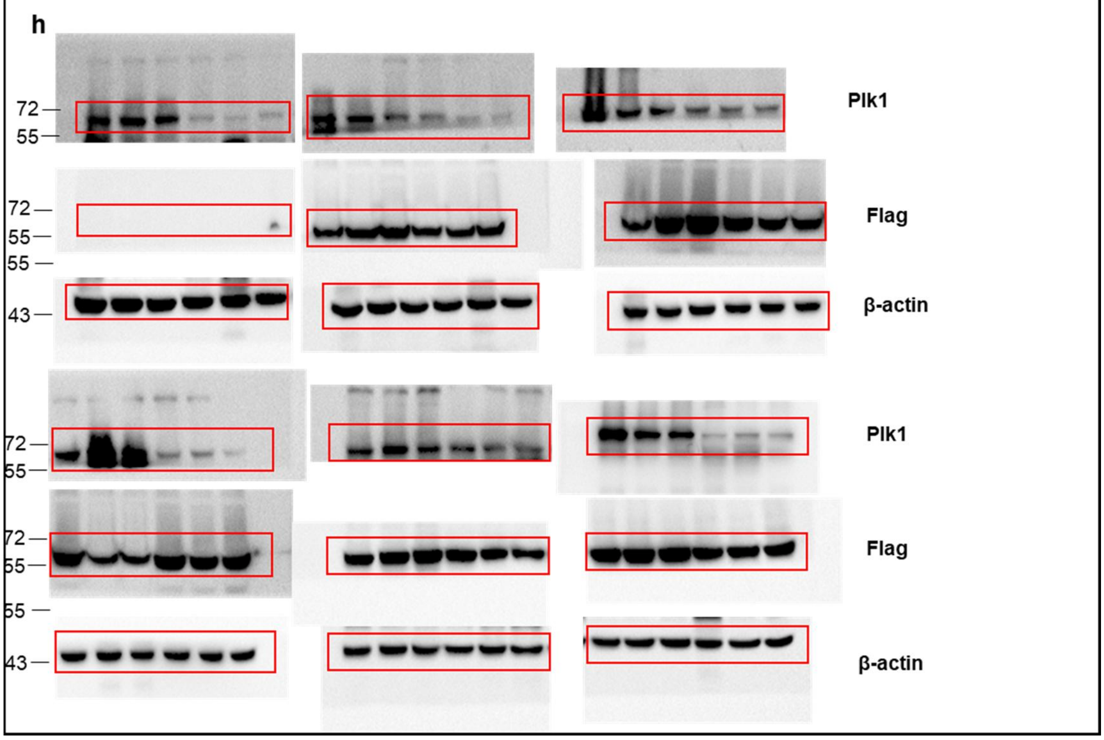

Supplementary Figure 8. The uncropped scans of immunoblots (continued).

Fig. S1

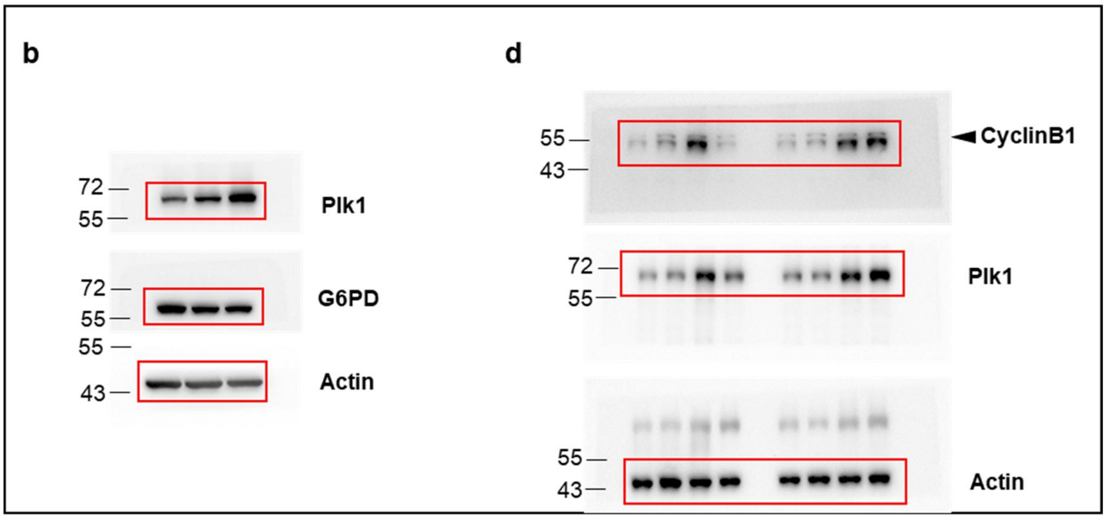

Fig. S2

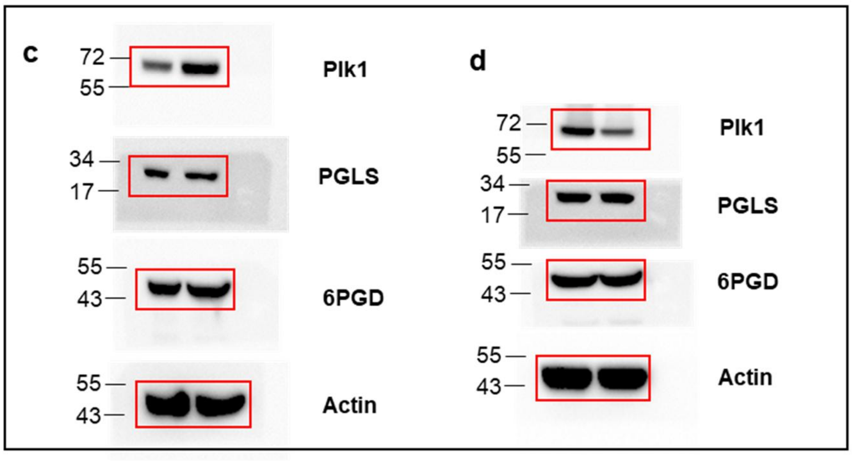

Supplementary Figure 8. The uncropped scans of immunoblots (continued).

Fig. S3

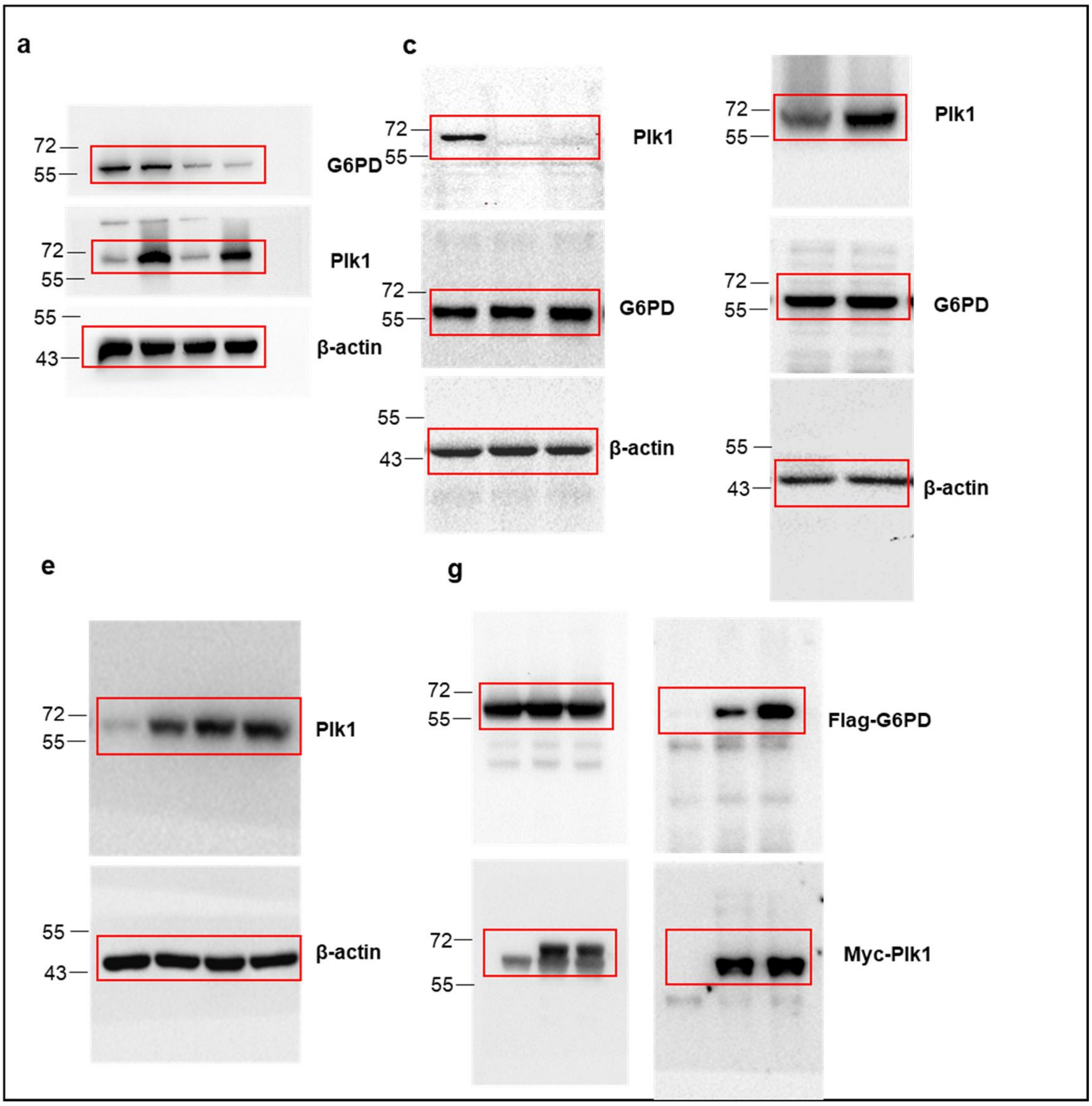

Supplementary Figure 8. The uncropped scans of immunoblots (continued).

Fig. S4

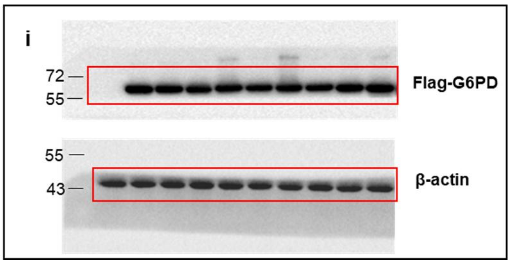

Fig. S5

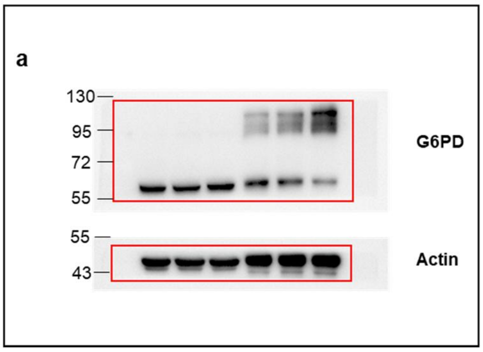

Fig. S7

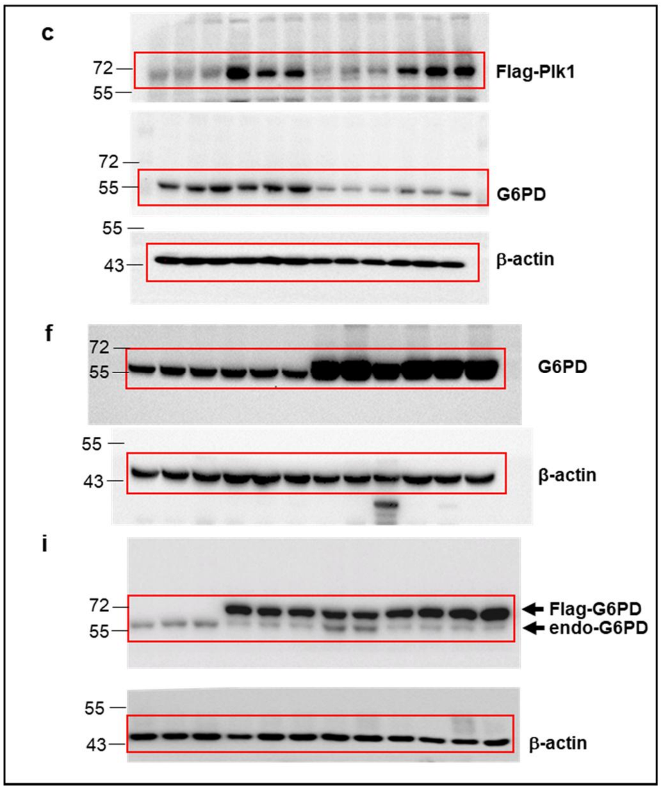

Supplementary Figure 8. The uncropped scans of immunoblots (continued).

Supplementary Table 1: Retention time and accurate m/z of each metabolite

Metabolite	RT	M/Z
R5P	5.701	299.0119 +/- 0.0025Da
AMP	5.046	346.056 +/- 0.010Da
dTMP	4.040	321.049 +/- 0.010Da
dCMP	5.517	306.050 +/- 0.005Da

Supplementary Table 2: Chemical shift of each metabolite

Compound	Group	Structure	δ(^{1}H)/ ppm	δ(^{13}C)/ ppm
R5P	β1	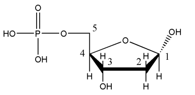	5.24	104.08
CXP	C1	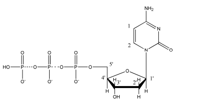	6.15	99.47
UXP	C1	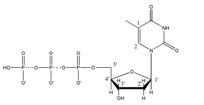	5.98	105.49
GXP	C1’	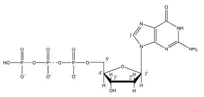	5.95	89.53
AXP	C1’	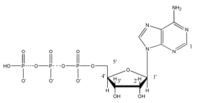	6.14	89.79
Lactate	C3	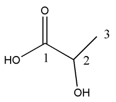	1.32	23.08
	C2		4.10	72.08
G6P	β2	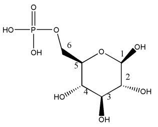	3.27	76.94
Pyruvate	C3	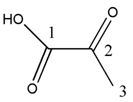	2.35	29.80

Supplementary Table 3: T1 experiments and calculation

298k, 10mM	ATP	ADP	AMP	CTP	CDP	CMP	GTP	GDP	GMP	UTP	UDP	UMP
T1	8.70	7.85	8.79	7.97	6.88	6.86	5.69	7.30	7.65	7.29	7.93	8.57
M(t)/M(0), * t=1.5s	0.16	0.17	0.16	0.17	0.20	0.20	0.23	0.19	0.18	0.19	0.17	0.16
SD		0.01			0.01			0.03			0.01	

* M(t) and M(0) are the magnetization at the time t and equilibrium state, t: relaxation delay.
